# Factors associated with motor manifestations in older adults with Alzheimer’s dementia: a cross-sectional analysis

**DOI:** 10.1007/s41999-025-01259-z

**Published:** 2025-07-21

**Authors:** Ioannis Liampas, Silvia Demiri, Polyxeni Stamati, Lefteris Lazarou, Christos Michailides, Chrysoula Marogianni, Antonia Tsika, Vasileios Siokas, Efthimios Dardiotis

**Affiliations:** 1 Department of Neurology, University Hospital of Larissa, Faculty of Medicine, School of Medicine, University of Thessaly, Mezourlo Hill, 41100 Larissa, Greece; 2 School of Medicine, University of Patras, 26504 Patras, Greece; 3 Department of Internal Medicine, University Hospital of Patras, 26504 Patras, Greece

**Keywords:** CDR, Cognitive performance, Depression, Neuropsychiatric symptoms, Angiotensin II receptor blockers, β-blockers

## Abstract

**Purpose:**

Motor signs are frequently observed over the clinical course of Alzheimer’s disease (AD). We explored the potential clinical associations of motor manifestations in AD.

**Methods:**

Our sample consisted of older adults (≥ 60 years) with AD from NACC. Individuals with Parkinson’s disease or other Parkinsonian syndrome or under anti-parkinsonian agents were excluded. UPDRS III was used to assess motor signs in nine domains: hypophonia; masked facies; resting tremor; action/postural tremor; rigidity; bradykinesia; impaired chair rise; impaired posture/gait; postural instability. A global motor variable assessed the presence of at least one motor sign. Binary logistic models were estimated for the global (primary) and individual motor domain variables (secondary outcomes).

**Results:**

A total of 4771 older, predominantly female, well-educated participants were analysed: 3556 without (75.4 ± 7.6 years, 45.6% males) and 1215 with motor manifestations (79.4 ± 7.8 years, 44.4% males). The most influential risk factor for motor manifestations in AD was the Clinical Dementia Rating stage: stage one increased the odds of motor signs by ~ 44%, stage two by ~ 168% and stage three by ~ 437%. Each additional point on the Geriatric Depression Scale elevated the odds of motor manifestations by ~ 5%, whereas each additional point on the Mini-Mental State Examination decreased these odds by ~ 2.5%. Cerebrovascular disease (by ~ 44%), diabetes mellitus (by ~ 25%), traumatic brain injury (by ~ 30%), alcohol abuse (by ~ 33%), anxiolytics (by ~ 36%), antidepressants (by ~ 31%), antipsychotics (by ~ 48%) and β-blockers (by ~ 33%) elevated the odds of motor manifestations. Angiotensin II receptor blockers decreased the odds of motor manifestations (by ~ 33%).

**Conclusion:**

Disease progression constitutes the most crucial clinical risk factor for motor manifestations in AD.

## Key summary points

**Aim:**

We explored the potential clinical associations of motor manifestations in Alzheimer’s dementia.

**Findings:**

Motor manifestations are related to disease progression, depression, cognitive impairment, comorbidities and medication intake.

**Message:**

Disease progression constitutes the most crucial clinical risk factor for motor manifestations in Alzheimer’s dementia.

## Introduction

Motor signs are frequently observed over the clinical course of Alzheimer’s disease (AD). Their underlying pathophysiology is not fully understood yet. Histopathologic changes including β-amyloid and tau pathology may either play a direct or an indirect role: cerebral atrophy might introduce motor manifestations by itself or instigate cognitive changes that mediate motor decline [1, 2]. Moreover, according to animal models, extracerebral β-amyloid depositions may expedite the progression of motor decline in AD [3]. Of note, mixed proteinopathies (coexisting non-AD neurodegenerative alterations), vascular changes, synaptic loss and cholinergic dysfunction are presumed to differentially contribute to the occurrence and exacerbation of these manifestations [1].

The prevalence of motor manifestations increases along with the severity of AD [4]. Some studies support that clinically important motor decline may even precede clinically relevant cognitive decline, co-occur with subtle neurocognitive changes, elevate the risk of incident AD or the risk of cognitive and non-cognitive adverse health outcomes over the AD continuum [5, 6]. At the same time, the management of these symptoms is often challenging and the armamentarium of pharmacological and non-pharmacological interventions remains ineffectual [1]. The burdensome nature, onerous management and prodigious healthcare impact (caregiver burden and financial cost) of motor manifestations in AD necessitate the identification of risk factors that may facilitate their prevention [7].

In the general population, psychotropic medications have been strongly related to motor symptoms and signs. Antipsychotics-especially first-generation antipsychotics- carry a great hazard for extrapyramidal symptoms [8], antidepressants have been associated with akathisia and tremor, among other movements disorders [9], benzodiazepines confer a risk towards ataxia especially in children [10], mood-stabilizing agents may introduce postural tremor in up to half of the treated individuals [11] and antiepileptic drugs could cause a variety of motor manifestations depending on their mechanism of action [12]. Motor symptoms also arise in the context of multiple systemic conditions. Cardiovascular risk factors elevate the risk of incident stroke and lead to more extensive cerebrovascular lesions which particularly contribute to lower extremity motor manifestations [13], diabetes mellitus (DM) can also lead to motor impairment via diabetic neuropathy [14] and hypertension may contribute to dilated perivascular spaces which relate to severe motor symptoms when located in the basal ganglia [15]. Traumatic brain injury (TBI) might feature with either short or long-term motor complications [16], depression can exacerbate bradykinesia [17] while anxiety can worsen tremor [18], B12 deficiency [19] or alcohol abuse [20] may lead to ataxia and illicit drug use could induce a series of movement disorders such as the introduction of dystonic reactions by cocaine [21].

The determinants of motor signs in AD remain largely unknown. To that end, we decided to investigate the potential clinical associations of motor manifestations in AD. For this purpose, we capitalized on data from the Uniform Data Set (UDS), a central repository of data, stewarded by the National Alzheimer's Coordinating Center (NACC) [22]. We focused on important clinical parameters, including common comorbidities and medication intake that have well-established risk properties in the general population.

## Methods

We aspired to explore the relationship of a number of clinical parameters, comorbidities and medications, with motor manifestations in older adults with AD, using data from the UDS. The UDS assembles standardized, prospectively collected, multidisciplinary data from multiple National Institute on Aging / National Institutes of Health (NIA/NIH)—funded Alzheimer's Disease Research Centers (ADRCs) across the United States [23].

## Methods

The UDS is freely available to research scientists upon request (https://naccdata.org/). The Institutional Review Boards overseeing each ADRC have approved study procedures before the initiation of the study. Signed consent was obtained from all participants (or surrogates) before participation. The objectives and key methodological features of the UDS have been detailly described elsewhere [24, 25]. In short, cognitively impaired and unimpaired volunteers are recruited based on each ADRC’s specific protocol. Uniform, standardized evaluations take place on an approximately annual basis. Among others, multidisciplinary assessments include family and personal medical history, clinical examinations, functional, neuropsychological and neuropsychiatric evaluations, and laboratory investigations in a minority of cases.

### Eligibility criteria and diagnostic procedure

The present study was based on NACC data from baseline assessments up to the December 2022 data freeze. Data were collected from a total of 46 ADRCs. Our sample consisted of older adults (≥ 60 years) with a diagnosis of AD. Those with a diagnosis PD or other parkinsonian syndrome as well as those treated with anti-parkinsonian agents were excluded. Cognitive diagnoses were established by either an interdisciplinary consensus team (in the majority of cases) or a single clinician (who conducted the examination), based on each ADRC’s specific protocol. Standard clinical criteria were applied for the diagnosis of dementia [26–29]. Imaging and/or cerebrospinal fluid (CSF) markers were available only in a minority of cases [30, 31].

### Measurement of motor signs based on the UPDRS-III

Unified Parkinson’s Disease Rating Scale Part III (UPDRS-III) comprises 27 subitems. We grouped these into nine motor domains as follows [32]: (1) hypophonia (single item); (2) masked facies (single item); (3) resting tremor (combined five items regarding tremor at rest in the face/lips/chin and four extremities); (4) action/postural tremor (combined two items regarding tremor at rest in the hands); (5) rigidity (combined five items regarding rigidity in the neck and four extremities); (6) bradykinesia (combined nine items: bilateral finger tapping, hand movements, rapid alternating movements of the hands, leg agility, and body bradykinesia); (7) impaired chair rise (single item); (8) impaired posture/gait (combined two items: posture and gait); and (9) postural instability (single item).

Each motor subitem was graded as absent (score < 2) or present (score ≥ 2). The rationale for this cutoff is based on the following: (1) this level of severity is more likely to be noted by the average clinician [4, 6]; and (2) a score of 1 is suggestive of a very mild motor change that could be observed with normal aging [33]. Then, we created nine dichotomous variables (motor domains), such that participants were said to have an abnormal motor sign if they scored ≥ 2 in at least one of the subitems of the respective motor domain. A global dichotomous variable was also created (presence of at least one motor sign), according to which participants were divided into those with (if they scored ≥ 2 in at least one motor domain) and those without (if they scored < 2 in all motor domains) motor manifestations. As described in more detail below (Results), different participant subsets were analysed per motor domain—according to the presence of missing data per motor assessment. To estimate the global motor variable, at least 6 out of the total 9 motor assessments had to be available for the respective participant.

### Covariates considered and exposures of interest

Our analyses were adjusted for the following potential confounders: participant’s age at the visit (in years) and education (in years of formal schooling)-both treated as scale variables-, sex (male/female), race (Caucasian, African American, Asian, other) and *apolipoprotein E* (*APOE*) genotypes (e3e3, e3e2, e3e4, e4e4, e2e4, e2e2) -all three treated as categorical variables-.

We emphasized the following important clinical parameters: global CDR (0.5, 1.0, 2.0, 3.0—as an index of symptoms’ severity), mini-mental state examination performance (MMSE—as a measure of cognitive impairment), geriatric depression scale scores (GDS—as a measure of depression), and a neuropsychiatric score (NPS—no, mild, moderate-severe neuropsychiatric symptoms—as a measure of neuropsychiatric burden). MMSE and GDS were treated as scale variables, CDR and NPS were treated as categorical variables. The NPS was calculated using data from the Neuropsychiatric Inventory Questionnaire (NPI-Q) (Cummings et al., 1994). NRI-Q evaluates 12 domains [delusions, hallucinations, agitation/aggression, depression/dysphoria, anxiety, elation/euphoria, apathy/indifference, disinhibition, irritability/lability, aberrant motor behaviour, night-time behaviours, and appetite/eating] according to a 4-point severity scale: no, mild (noticeable, but not a significant change); moderate (significant, but not a dramatic change); or severe (very marked or prominent; a dramatic change). For each NPI-Q domain, participants were grouped into three categories: 0: absent; 1: mild; 2: moderate and severe symptomatology (due to the small prevalence of moderate and severe symptoms) [35]. Sequentially, the composite NPS was calculated as follows: 0: no symptoms; 1: at least one mild symptom with no moderate and/or severe symptoms, 2: at least one moderate and/or severe symptom.

We emphasized the following comorbidities (treated as dichotomous categorical variables): history of traumatic brain injury (TBI), vitamin B12 deficiency, alcohol or other substance abuse (with clinically significant impairment occurring over a 12-month period manifested in one of the following areas: work, driving, legal, or social), current smoking, diabetes mellitus (DM), hypertension, hypercholesterolemia, cardiovascular (CaVD; heart attack/cardiac arrest or coronal angioplasty/endarterectomy/stent or cardiac bypass procedure or congestive heart failure or atrial fibrillation or implanted pacemaker) and cerebrovascular disease (CeVD; stroke or transient ischemic attack). These comorbidities were positively assessed according to participant or co-participant reporting of either recent/active or remote/inactive conditions. For DM and hypercholesterolemia, the current use of antidiabetics and lipid-lowering agents was additionally considered, respectively.

Finally, we emphasized the following medications (treated as dichotomous categorical variables): current use of antidepressant [including selective serotonin reuptake inhibitor (SSRIs), serotonin and norepinephrine reuptake inhibitors (SNRIs), tricyclic, tetracyclic, monoamine oxidase inhibitors (MAOIs), miscellaneous (e.g., bupropion, trazodone, mirtazapine, 5-hydroxytryptophan,)], anxiolytic [including benzodiazepines, barbiturates, and other sedatives and hypnotics (e.g., buspirone, hydroxyzine, zolpidem, melatonin)] and antipsychotic agents [including phenothiazines, thioxanthenes, atypical antipsychotics, combinations with antipsychotics (e.g., amitriptyline-chlordiazepoxide or perphenazine) and other miscellaneous agents (e.g., lithium)], FDA-approved medication for AD (tacrine, donepezil, rivastigmine, galantamine and memantine) and antihypertensive medication [five separate variables, i.e., diuretics (yes/no), calcium channel blockers (CCBs, yes/no), β-blockers (yes/no), angiotensin converting enzyme inhibitors (ACEis, yes/no) and angiotensin II receptor blockers (ARBs, yes/no)].

### Statistical analysis

Baseline differences between those with and without any motor signs were analysed using (1) independent samples t-test (scale variables) and (2) Pearson’s chi-squared tests (categorical variables). The conventional threshold of *α* = 0.05 was implemented.

Associations between the presence of at least one motor sign and exposures of interest were estimated using binary logistic regression (primary outcome). A backward conditional model with a probability for stepwise entry and removal at *α* = 0.05 was applied. Separate binary logistic regression models were tested for each motor domain (secondary outcomes). Due to the number of exploratory analyses (9 in total) the probability threshold for the exploratory backward conditional models was lowered at *α* = 0.005 (entry and removal). Finally, the main analysis was reproduced in those ≥ 80 years of age to examine if the same pattern of associations is to be expected in the oldest old.

The statistical analysis was performed using the IBM SPSS Statistics Software Version 27 (Chicago, IL, USA). Effect sizes (odds ratios, ORs) and precision estimates (95% confidence intervals, 95%CIs) are presented.

## Results

### Participant characteristics and missing data

**Fig. 1:**
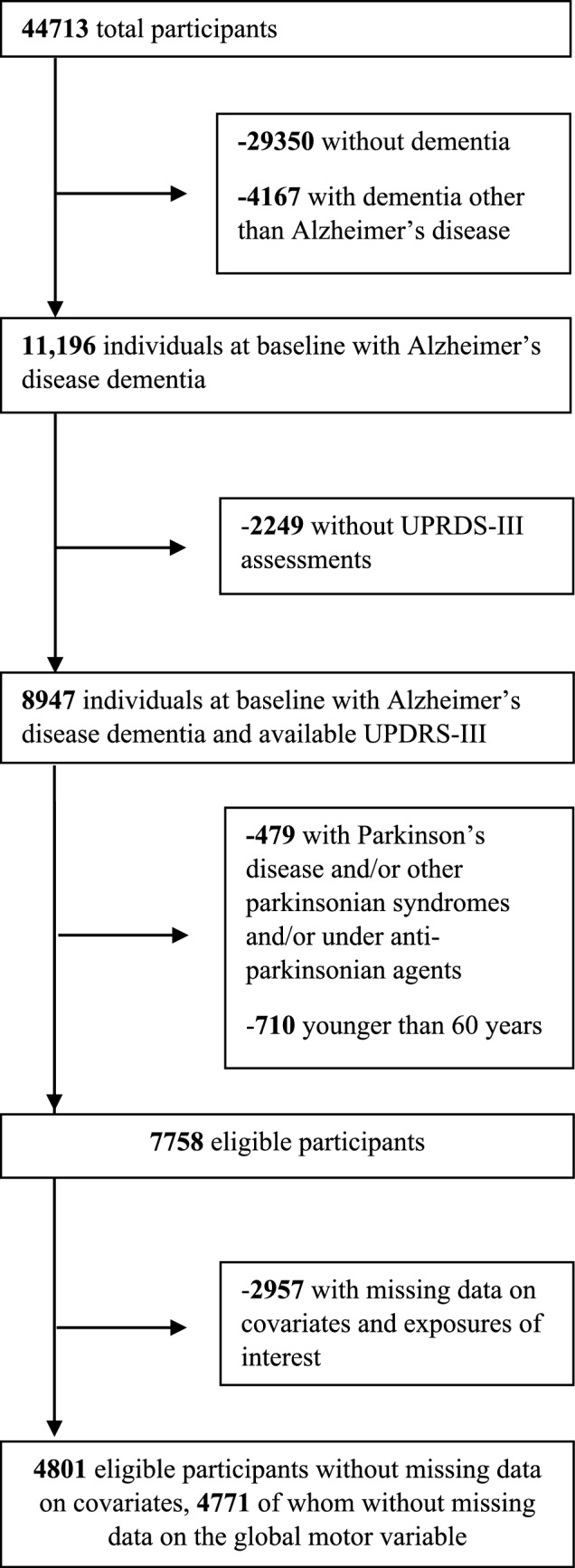
Participant flowchart

The beginning database included 44,713 individuals with at least one UDS evaluation. Among them, 15,363 individuals were diagnosed with dementia and 11,196 of them with AD. Motor symptoms were assessed using the UPDRS-III in the first two versions of the UDS; thus, 2249 participants evaluated on the 3rd version were excluded. After the exclusion of those with a diagnosis of PD or other parkinsonian syndrome, those on anti-parkinsonian medication and those under the age of 60 years, 7758 participants were eligible for the present study (Fig. 1). Among eligible participants, there were 2957 with missing data on at least one of the covariates (mostly on APOE, *N* = 1948) and/or exposures of interest. Among eligible participants without missing data on covariates (*N* = 4801), a total of 30 participants had missing data on the global motor variable (*N* = 4771 individuals were analyzed for the global motor variable); 30 had missing data on resting tremor and rigidity, 32 on hypophonia, masked facies, action-postural tremor and bradykinesia, 52 on impaired posture-gait, 61 on impaired chair rise and 152 on postural instability. Therefore, slightly different participant subsets were analysed per motor domain.

Participant characteristics are in Table 1. In brief, those with at least one motor sign (*N* = 1215) were older and less educated than those without any motor signs (*N* = 3556). Sex and race distributions were similar between the two groups. APOE genotypes differed between the two groups with e3e4 and e4e4 genotypes being more prevalent among those without motor signs and e3e3 and e3e2 being more common among those with at least one motor sign. Regarding exposures of interest, those with motor signs performed worse on MMSE and had greater GDS, NPS and CDR scores. Among comorbidities, CeVD, CaVD, DM, hypertension, B12 deficiency and alcohol abuse were more common in those with motor signs. As for, medication intake, the use of ARBs was more frequent among those without any motor signs, whereas anxiolytics, antidepressants, antipsychotics, β-blockers, diuretics and ACEis intake was more prevalent in those with motor signs.

**Table 1:** Participant characteristics

Variable	*No motor sign* (*N* = 3556)	*At least one motor sign* (*N* = 1215)	*p*-value
Age (years)	75.4 ± 7.6	79.4 ± 7.8	*p* < 0.001
Sex (male/%)	1620 /45.6%	540 /44.4%	*p* = 0.505
Education (years)	14.4 ± 3.6	13.8 ± 3.8	*p* < 0.001
Race (Caucasian / African American / Asian / other)	3021/411/41/83 (85.0/11.5/1.2/2.3%)	1021/140/17/37 (84.0/11.5/1.5/3.0%)	*p* = 0.500
*APOE* genotype (e3e3/e3e4/e3e2/ e4e4/e4e2/e2e2)	1225/1557/143 /515/110/34 (34.4/43.8/4.0 /14.5/3.1/0.2%)	492/477/78 /131/34/3 (40.5/39.3/6.4 /10.8/2.8/0.2%)	*p* < 0.001
MMSE (30)	21.5 ± 5.1	19.5 ± 5.8	*p* < 0.001
GDS (15)	2.3 ± 2.5	2.7 ± 2.8	*p* < 0.001
NPS (none/mild/ moderate or severe)	601/1258/1697 (16.9/35.4/47.7%)	176/386/653 (14.5/31.8/53.7%)	*p* = 0.001
Global CDR (0.5/1.0/2.0/3.0)	1382/1744/394/36 (38.9/49.0/11.1/1.0%)	265/581/305/65 (21.8/47.8/25.1/5.3%)	*p* < 0.001
TBI (Yes /%)	334 /9.4%	131 /10.8%	*p* = 0.162
CaVD (Yes /%)	757 /21.3%	337 /27.7%	*p* < 0.001
CeVD (Yes /%)	314 /8.8%	187 /15.4%	*p* < 0.001
Smoking (Yes /%)	141 /4.0%	44 /3.6%	*p* = 0.667
Diabetes mellitus (Yes /%)	430 /12.1%	188 /15.5%	*p* = 0.003
Hypertension (Yes /%)	1847 /51.7%	706 /58.1%	*p* < 0.001
Hypercholesterolemia (Yes /%)	2086 /58.7%	687 /56.5%	*p* = 0.201
B12 deficiency (Yes /%)	213 /6.0%	93 /7.7%	*p* = 0.049
Alcohol abuse (Yes /%)	201 /5.7%	93 /7.7%	*p* = 0.015
Other substance abuse (Yes /%)	22 /0.6%	6 /0.5%	*p* = 0.828
Anxiolytics (Yes /%)	277 /7.8%	126 /10.4%	*p* = 0.006
Antidepressants (Yes /%)	1238 /34.8%	487 /40.1%	*p* = 0.001
Antipsychotics (Yes /%)	125 /3.5%	99 /8.1%	*p* < 0.001
FDA approved drugs for AD (Yes /%)	2536 /71.3%	861 /70.9%	*p* = 0.764
ACEis (Yes /%)	671 /18.9%	275 /22.6%	*p* = 0.005
ARBs (Yes /%)	345 /9.7%	92 /7.6%	*p* = 0.028
β-blockers (Yes /%)	705 /19.8%	309 /25.4%	*p* < 0.001
CCBs (Yes /%)	546 /15.4%	203 /16.7%	*p* = 0.273
Diuretics (Yes /%)	510 /14.3%	228 /18.8%	*p* < 0.001

Scale variables are presented in mean ± standard deviation; categorical variables are presented in absolute numbers (proportions); *N* number of individuals, *APOE* *apolipoprotein*, *MMSE* mini-mental state examination, *GDS* geriatric depression scale, *NPS* neuropsychiatric score, *CDR* clinical dementia rating scale, *TBI* traumatic brain injury, *CaVD* cardiovascular disease, *CeVD* cerebrovascular disease, *AD* Alzheimer’s disease, *ACEis* angiotensin converting enzyme inhibitors, *ARBs* angiotensin II receptor blockers, *CCBs* calcium channel blockers

### Determinants of motor manifestations in AD

**Fig. 2:**
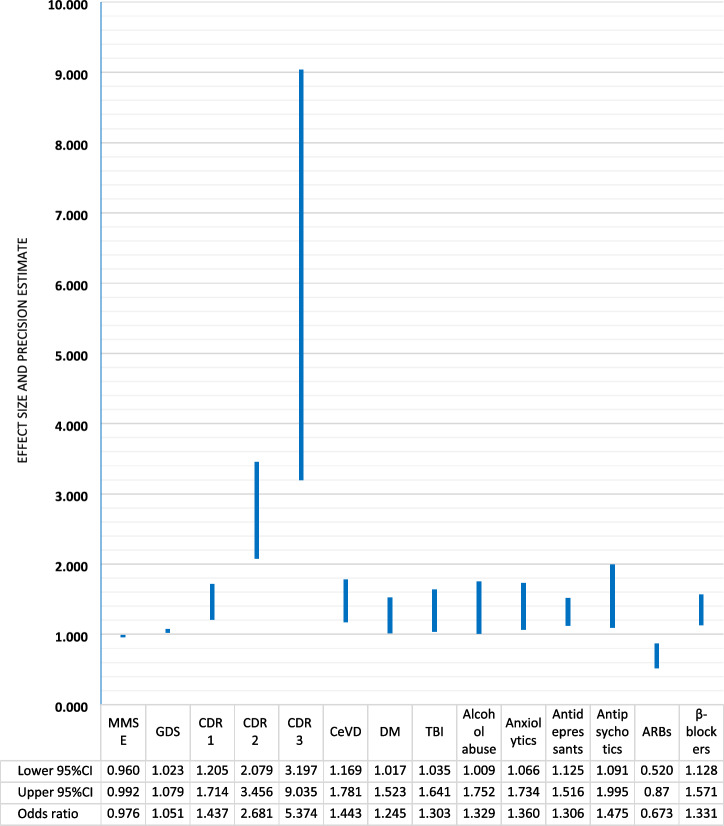
Determinants of motor manifestations in Alzheimer's disease dementia

The risk factors related to motor signs in AD, the sizes of the estimated associations and their precision, are in Fig. 2. The most influential risk factor for motor manifestations in AD was the CDR stage (reflecting symptoms’ severity). In specific, stage one increased the relevant odds by ~ 44%, stage two by ~ 168% and stage three by more than 4 times. Each additional point on the GDS increased the odds of motor signs by ~ 5%, whereas each additional point on the MMSE decreased these odds by ~ 2.5%. Among comorbidities, CeVD (by ~ 44%), DM (by ~ 25%), history of TBI (by ~ 30%) and alcohol abuse (by ~ 33%) elevated the odds of motor manifestations. As for medication intake, anxiolytics (by ~ 36%), antidepressants (by ~ 31%), antipsychotics (by ~ 48%) and β-blockers (by ~ 33%) conferred increased odds towards motor signs, whereas ARBs decreased the relevant odds (by ~ 33%).

**Fig. 3:**
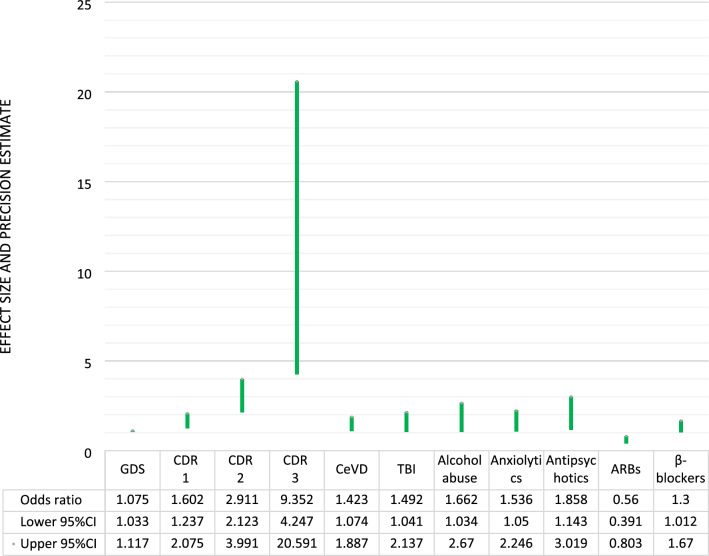
Determinants of motor manifestations in Alzheimer's disease dementia among individuals ≥80 years of age

Subgroup analysis involving the oldest old (those ≥ 80 years, 1765 individuals) reproduced the majority of the aforementioned findings (Fig. 3). Again, CDR stage was the most influential parameter and the same pattern of associations was replicated for GDS, CeVD, TBI, Alcohol abuse and intake of anxiolytics, antipsychotics, ARBs and β-blockers. On the other hand, MMSE, DM and antidepressants were not found significant in this subgroup.

With respect to specific motor manifestations, the CDR stage was again the most important risk factor increasing the odds of every motor manifestation (Table 2). The relevant odds exhibited progressive enlargement with more advanced disease severity. Stages two and three exhibited the most marked risk-conferring properties in relation to any other risk factor, in the context of every motor manifestation. Of note, disease severity was the only robust risk factor associated with tremor (resting, action-postural) and hypophonia. Depression was found to increase the odds of postural instability, impaired posture-gait and impaired chair rise; CeVD elevated the odds of postural instability, impaired posture-gait, impaired chair rise and bradykinesia; and DM enlarged the odds of impaired posture-gait, impaired chair rise and bradykinesia. CaVD and TBI conferred increased odds towards impaired chair rise. As for pharmaceutical agents, β-blockers were related to postural instability, diuretics to impaired chair rise, antidepressants to bradykinesia and antipsychotic agents to both bradykinesia, rigidity, impaired posture-gait and masked facies.

**Table 2:** Determinants of specific motor signs in Alzheimer’s disease

Motor signs	CDR 1	CDR 2	CDR 3	GDS	CeVD	CaVD	DM	TBI	Antidepressants	Antipsychotics	Diuretics	β-blockers
Hypophonia	N/A	10.57 (4.00, 27.94), * p* < 0.001	57.35 (20.26, 162.35), * p* < 0.001	N/A	N/A	N/A	N/A	N/A	N/A	N/A	N/A	N/A
Masked facies	N/A	3.77 (1.92, 7.40), * p* < 0.001	15.26 (6.91, 33.69), * p* < 0.001	N/A	N/A	N/A	N/A	N/A	N/A	3.47 (2.01, 6.01), * p* < 0.001	N/A	N/A
Resting tremor	N/A	3.64 (1.99, 6.65), * p* < 0.001	N/A	N/A	N/A	N/A	N/A	N/A	N/A	N/A	N/A	N/A
Action-postural tremor	N/A	2.48 (1.72, 3.58), * p* < 0.001	N/A	N/A	N/A	N/A	N/A	N/A	N/A	N/A	N/A	N/A
Rigidity	1.67 (1.12, 2.36), * p* = 0.003	4.08 (2.78, 5.95), * p* < 0.001	7.56 (4.22, 13.54), * p* < 0.001	N/A	N/A	N/A	N/A	N/A	N/A	1.91 (1.24, 2.92), * p* = 0.003	N/A	N/A
Bradykinesia	1.81 (1.38, 2.38), * p* < 0.001	4.17 (3.08, 5.65), * p* < 0.001	10.85 (6.70, 17.55), * p* < 0.001	N/A	1.61 (1.22, 2.12), * p* = 0.001	N/A	1.55 (1.19, 2.01), * p* = 0.001	N/A	1.41 (1.15, 1.74), * p* = 0.001	1.70 (1.19, 2.43), * p* = 0.004	N/A	N/A
Impaired chair rise	2.26 (1.64, 3.13), * p* < 0.001	4.16 (2.90, 5.97), * p* < 0.001	12.73 (7.22, 22.43), * p* < 0.001	1.12 (1.08, 1.16), * p* < 0.001	1.74 (1.30, 2.34), * p* < 0.001	N/A	1.65 (1.22, 2.23), * p* = 0.001	N/A	N/A	N/A	1.58 (1.21, 2.07), * p* = 0.001	N/A
Impaired posture-gait	N/A	3.66 (2.56, 5.24), * p* < 0.001	7.56 (4.12, 13.73), * p* < 0.001	1.09 (1.05, 1.14), * p* < 0.001	1.94 (1.44, 2.62), * p* < 0.001	1.52 (1.17, 1.97), * p* = 0.002	1.90 (1.39, 2.60), * p* < 0.001	1.78 (1.24, 2.57), * p* = 0.002	N/A	2.13 (1.40, 3.25), * p* < 0.001	N/A	N/A
Postural instability	N/A	2.35 (1.66, 3.34), * p* < 0.001	6.58 (3.58, 12.01), * p* < 0.001	1.13 (1.08, 1.17), * p* < 0.001	2.09 (1.54, 2.83), * p* < 0.001	N/A	N/A	N/A	N/A	N/A	N/A	1.56 (1.19, 2.04), * p* = 0.001

Odds ratios with 95% confidence intervals and *p*-values are provided; associations were significant according to the *α* = 0.005 threshold; *N/A* non-applicable, i.e., non-significant association according to the *α* = 0.005 threshold; *MMSE* mini-mental state examination, *GDS* geriatric depression scale, *NPS* neuropsychiatric score, *CDR* clinical dementia rating scale, *TBI* traumatic brain injury, *CaVD* cardiovascular disease, *CeVD* cerebrovascular disease, *AD* Alzheimer’s disease, *ACEis* angiotensin converting enzyme inhibitors, *ARBs* angiotensin II receptor blockers, *CCBs* calcium channel blockers

## Discussion

The present study revealed that the most prominent risk factor for motor manifestations in AD is disease severity, reflected in CDR. The direction of this relationship can be presumed due to the presence of a ‘‘dose–response’’ association. Every motor sign -with the potential exception of tremor- exhibited progressively increased odds of occurring with greater disease severity. Stages two and three exhibited the most remarkable risk-conferring properties, compared to the entirety of the investigated parameters. Similarly, previous descriptive evidence concurs that the prevalence of motor signs escalates with AD progression and that the rates of motor decline are more remarkable for speech/facial expression and relatively unremarkable for tremor [4]. These findings also align with the prevailing theory that connects motor manifestations to the neuropathological alterations of the disease [1, 3].

Better cognitive performance was related to decreased odds of motor manifestations. Motor manifestations have been associated with cognitive performance in both cognitively unimpaired and impaired individuals (mild cognitive impairment and AD) [2, 5, 36]. Growing evidence indicates that the most critical domains of cognition that interfere with motor function are executive function, visuospatial perception and attention, which relies on executive control, orientation and alertness [37]. In the setting of neurodegeneration, neuronal loss within these networks might introduce motor manifestations mediated by cognitive rather than-directly-motor control impairment [38]. Intriguingly, depression may also manifest with slower mental processing (component of attention) and working memory deficits (mediated by executive control and attention) which could at least partly explain the relationship between depression and motor dysfunction [39, 40]. Of course, the cross-sectional nature of the current analysis implies that motor dysfunction could also be responsible for increased levels of depression and worse cognitive performance, and not the other way around.

Regarding comorbidities and medication intake, CeVD, CaVD, DM, history of TBI, alcohol abuse, anxiolytics, antidepressants, and antipsychotics conferred increased odds of motor signs. Of note, antipsychotics were prominently related to most Parkinsonian features (including masked facies, bradykinesia and rigidity). These associations extend back to normal cognition and the discussion of the underlying mechanisms exceeds the objectives of the current study [8–10, 14, 20, 41, 42]. Of interest, antihypertensive agents were also related to motor signs. Diuretics and β-blockers were associated with elevated odds, whereas ARBs were linked to reduced odds. β-blockers and diuretics have been related to orthostatic hypotension [43, 44] and muscular weakness [45, 46]; these associations may also explain their relationship with motor signs, postural instability and impaired chair rise in particular. On the other hand, the direction of our associations may be the opposite, suggesting that individuals with motor manifestations are more likely to be prescribed these medications. The same applies to ARBs; however, the neuroprotective properties and beneficial effects of ARBs on cognition are well-established and a direct or indirect (mediated via cognition) effect on motor function cannot be excluded [47–49].

Our study has several strengths, notably the large sample of older individuals with AD. The extensive assessments of the UDS allowed us to explore a large number of important clinical parameters in the analytical part of the article: AD stage, cognitive performance, depression scores, neuropsychiatric burden, comorbidities and medications [50]. This analysis has several weaknesses, as well. First, in the vast majority of NACC participants, the diagnosis of the major neurocognitive entities was based on clinical criteria; objective biomarkers were not uniformly available. Therefore, some dementia cases were probably erroneously misclassified as AD (as well as the other way around). Second, although we analysed a large number of important clinical parameters, our findings may have been driven by residual confounding (it is not be possible to capture the effect of every potential confounder) or the non-trivial proportion of missing data. Third, motor assessments were based on the UPDRS-III, a widely used tool in clinical and research settings; regardless, some variability should be anticipated among different assessors in the quantification of motor signs. Moreover, the use of the UPDRS-III tool confined our investigations, in terms of the spectrum of motor assessments. In other words, UPDRS III is particularly focused on Parkinsonian signs, whereas very little information is collected on general motor manifestations and/or cerebellar signs. Finally, the low prevalence of certain motor signs (especially hypophonia, resting tremor and masked facies) might have underpowered some aspects of our analysis, precluding the unravelling of several potentially non-trivial associations.

## Conclusion

AD severity constitutes the most crucial clinical risk factor for motor manifestations. Depression, cognitive performance, CeVD, CaVD, DM, TBI, alcohol abuse, anxiolytics, antidepressants, antipsychotics, diuretics, β-blockers and ARBS are also related to motor signs, but the direction of the association requires further research. Future studies ought to investigate these associations in a longitudinal fashion examine if the management of modifiable risk factors could alleviate the prevalence and severity of motor manifestations and expand motor assessments to involve additional symptoms and signs, beyond Parkinsonian manifestations.

## Data Availability

For further information on access to the NACC database, please contact NACC (contact details can be found at https://naccdata.org/).
